# Diaspora Policies, Consular Services and Social Protection for French Citizens Abroad

**DOI:** 10.1007/978-3-030-51245-3_11

**Published:** 2020-10-31

**Authors:** Jean-Thomas Arrighi, Jean-Michel Lafleur

**Affiliations:** 1 FRS-FNRS & Centre for Ethnic and Migration Studies (CEDEM), University of Liege, Liege, Belgium; 2 Centre for Ethnic and Migration Studies (CEDEM), University of Liege, Liege, Belgium; 3 University of Neuchâtel, Neuchâtel, Switzerland; 4 FRS-FNRS & Centre for Ethnic and Migration Studies (CEDEM), University of Liege, Liege, Belgium

## Abstract

While predominantly a country of immigration, France also counts with a sizeable population of citizens abroad of around three million individuals (4% of the domestic population). This chapter provides a general overview of France’s diaspora institutions, consular policies and social protection policies for citizens abroad. It describes in detail expatriates’ conditions of eligibility and access to welfare in the areas of unemployment, health care, pensions, family benefits and economic hardship. It shows that France, by European standards, has a comparatively strong level of engagement with its expatriates, particularly in the areas of electoral rights, culture and social protection. This must be understood in the light of France’s colonial history, its continued ambition to be a global actor, and its well-developed domestic welfare state that has increasingly become de-territorialised.

## Introduction

This chapter presents the general overview of France’s policies towards its citizens abroad, with a focus on social protection policies.^1^ In spite of policy-makers’ focus on immigration, France has also experienced considerable emigration in recent decades. Following a brief presentation of the country’s diaspora infrastructure, we introduce its key diaspora engagement policies, with a particular focus on consular policies and France’s extensive external franchise, regarding both expatriates’ electoral participation as well as their political representation in the French Parliament. We then discuss in detail the conditions of eligibility and access to social protection for non-resident citizens in the areas of unemployment, healthcare, pensions, family benefits and economic hardship. Overall, the chapter argues that, as a result of its colonial history, its continued ambition to be a global actor and its well-developed welfare state, France has developed a comparatively strong form of engagement with its citizens abroad in the area of welfare.

## Diaspora Characteristics and Home Country Engagement

### The French Diaspora and Its Relations with the Homeland

Historically, France has been rather a country to which one comes than one from which one leaves. This is reflected in migration statistics, with inflows significantly exceeding outflows (Brutel 2015). Yet, emigration has increased over the past 20 years. The number of French passport holders around the world is hard to assess, especially since the French nationality law allows for dual citizenship and does not place restriction upon the transmission of citizenship through *jus sanguini* of citizens born abroad of at least one French parent. According to recent estimates, the number of French citizens abroad should range between two to three million (Brutel 2015).

The Ministry of Interior collects and provides up-to-date information on the number of French citizens abroad who are duly registered in consular population registries. In 2017, there were 1.8 million French citizens who were registered in French consulates abroad, 37% of whom resided in another Member State of the European Union (EU). The five main countries of destination of French expatriates all share geographical borders with France: the United Kingdom (UK- 147,000), Belgium (128,000), Germany (117,000), Spain (85,000), and Italy (78,000).^2^ The overall proportion of men and women in the population of French citizens abroad was roughly equal in 2017, in spite of significant variations across regions. Women represented 42.5% of the registered population in Asia-Oceania, against 53.4% in the EU. As for the age distribution, 34% were less than 25 years old, 51% between 25 and 60, and 15% over 60 years old.^3^ While there is little information on the socio-economic characteristics of this population, a 2014 study carried out by the Chamber of Commerce of the Paris region noted that French citizens abroad generally have a significantly higher level of education than the domestic population, with 12% of them holding a doctorate degree and an additional 37%, a higher education degree. Among other findings, the survey also showed that 57% of the respondents declared earning more than EUR 30,000 per year (Biacabe and Robert 2014: 13).

By European standards, emigration from France is a relatively recent phenomenon, as comparatively few people left the country in the nineteenth century. This led some historians to argue that the limited size of the French diaspora in the United States (US) in the first half of the twentieth century seriously harmed France’s geostrategic interests, especially when compared to Germany (Haglund 2012). To this day, the lack of cohesion and political mobilisation among French nationals abroad has remained a pervasive fact and contrasts sharply – as we show below – with the sustained efforts and considerable resources that have been invested by French authorities to reach out to them during the past two decades. Hence, while it can be argued that, in a sociological sense, there is no such thing as a French diaspora, France has deployed a variety of diaspora-building policies. Hence, in accordance with Roger Brubaker’s well-known distinction, the French diaspora should rather be understood as a top-down category of political practice than a bottom up category of analysis (Brubaker 2005).

### Diaspora Infrastructure

As mentioned in the introduction to this volume (Lafleur and Vintila this volume), France’s diaspora infrastructure is comparatively more extensive when compared to other EU Member States. In recent years, France has engaged in a far-reaching diaspora-building project through a variety of institutions: ministries, the diplomatic network, consultative and representative bodies, and parliamentary representation. French political parties have also significantly increased their presence abroad in recent years.

As far as the executive branch of Government is concerned, the responsibility for French citizens abroad falls under the remit of the French Ministry for Europe and Foreign Affairs (*Ministère français pour l’Europe et les affaires étrangères*). Until recently, there was a sub-ministry for French Abroad (*Secrétaire d’état pour les Français de l’étranger*), located within the Ministry for Foreign Affairs. It was not renewed under the presidency of Emmanuel Macron after he took office in May 2017. In their countries of residence, French expatriates can count on France’s vast diplomatic network – the third largest in the world, after the US and China –, which includes 213 consulates and over 500 diplomatic missions. There are about 30 countries where France has no official diplomatic representation. For most of these countries, there are ad-hoc arrangements to ensure access to consular services. For instance, French citizens in Bhutan depend upon the French consular representation in India. The status of French honorary consuls is regulated under Decree 76–548 of 1976. In terms of services to French abroad, the main difference with ordinary consuls is that they cannot deliver visas to foreigners wishing to travel to France. Honorary consuls are not paid and do not have to hold French nationality. They are appointed by the French Minister of Foreign affairs and their core mission is to ensure the protection of the interests of French citizens abroad.

Second, France has long established formal forums to reach out to its population abroad through consultative bodies that meet either in France or in the countries of residence. In 1948, an official decree created the High Council for French Abroad (*Conseil supérieur des Français de l’étranger*), which was replaced in 2004 by the Assembly for French Abroad (*Assemblée des Français de l’étranger,* AFE), which issues non-binding opinions to French authorities on issues of relevance to French citizens abroad.

The Consular Council (*Conseil consulaire*) is the institution responsible for defending the interests of French expatriates in their countries of residence. It is a consultative body dedicated to consular affairs. Since 2013, its members are elected every 6 years by citizens abroad officially registered in each of the 130 consular constituencies. Nine councillors sit in each Consular Council. As shown in this volume, among EU Member States, only Italy has a similar form of representation at the consular level. Their task consists in assisting the administration by issuing an opinion on all matters of interest to French citizens residing within the constituency, and they play a prominent role in the delivery of social entitlements to citizens abroad (see below).

Third, as part of its policy of enfranchising citizens abroad, France has opted for a discrete representation through reserved seats in the Lower and Upper Chambers of the national Parliament. For the election of the Upper Chamber (*Assemblée Nationale*)*,* there are 11 geographically-defined extra-territorial constituencies, based upon the demographic distribution of French citizens abroad (since 2011). In the Lower Chamber (*Sénat*), there are 12 reserved seats for representatives of citizens abroad, but these are non-geographically defined as those representatives are indirectly elected by consular councillors. This presence in the two Chambers guarantees the representation of emigrants’ interests in the French Parliament.

Fourth, and mainly as a result of successive reforms strengthening the political participation and representation of expatriates during the past decade, French political parties have also developed their presence abroad. With the introduction of extra-territorial constituencies, all mainstream parties have created ‘federations’, ‘committees’ or ‘sections’ abroad.^4^ Most of those extra-territorial sections are often empty shells that are reactivated and play a role in the campaign prior to national or European elections. However, party sections abroad provide candidates with the opportunity to gain international visibility and polish their international stature. More importantly, the creation of an extra-territorial constituency with reserved seats has created an emerging class of ‘extra-territorial’ political entrepreneurs, who specifically target voters residing in specific countries for elections (see also Pellen 2013; Kernalegenn and Pellen 2020).

A number of French cultural institutions are also present abroad. Their mission is presented in the next section as they are closely tied to cultural policies, one of France’s core area of engagement abroad. In 2018, France introduced the so-called Support Fund to the Network of French Associations Abroad (STAFE, *Dispositif de soutien au tissu associatif des Français à l’étranger*). In its first year, it was endowed with EUR 2 million, which were distributed to relatively small associations in a range of countries. While such policy is relatively rare, it can also be found in other countries covered in this volume, such as Ireland and Poland (see, for instance, Hickman this volume).

### Key Engagement Policies

France has a very elaborated and diversified set of diaspora policies, though the political aspect stands out. By international standards, France has one of the most generous extra-territorial franchise for citizens residing abroad (Arrighi 2014, 2018). They may participate in presidential elections, European elections and national referendums. Since 2012, they may even directly elect their own representatives to the National Assembly, where 11 seats out of 577 are reserved to the representation of ‘French citizens residing outside of France’. In addition, their interests are indirectly represented through 12 reserved seats in the Senate. In some aspects, electoral registration is easier for French expatriates than for residents. Indeed, consular electoral registries are automatically generated from the population registries administered by consulates. Furthermore, French expatriates can cast a ballot from abroad either directly in the consular or diplomatic premises of their constituency of residence or by appointing a proxy. In parliamentary elections, they may even cast an electronic vote.^5^

When it comes to basic consular services, France’s policies are not significantly different from those observed in other EU Member States discussed in this volume. French citizens abroad may renew their national identity card (*carte nationale d’identité*) or passport in consulates abroad. The procedure is quite straightforward, especially given the gradual digitalisation of administrative procedures. Applicants must submit an existing ID document (even if expired), justification of residence abroad and, in the event that the existing document is not ‘secured’ (e.g. biometric passport), a birth certificate. The birth certificate usually can be downloaded online by the consular administration. The renewal of an ID is free of charge, but the passport renewal has an administrative cost of EUR 92 (less for a minor).

France also offers repatriation and assistance to French citizens abroad in the event of death, illness, or hardship. Under “extraordinary circumstances”, the state can organise the repatriation and pay related expenses, although the beneficiary’s family is expected to fully reimburse those expenses. Those circumstances may include cases of French citizens abroad facing economic hardship, though the lack of clear criteria in the legislation leaves the relevant consular authorities with significant discretionary power in assessing individual cases. Once they are declared eligible for repatriation, individuals are then directed by authorities towards associations in France, which may cater for their particular needs. Upon their return to France and provided they meet the general eligibility requirements, they may be entitled to a provisional shelter, medical assistance, or the State Medical Aid (*Aide Médicale de l’État)*, a universal health coverage for persons who are not affiliated to the general regime. According to a 2017 report commissioned by the Prime Minister (*Rapport du Gouvernement sur la situation des Français établis hors de France*), the Ministry spent almost 500,000 euros in 2016 in organizing 237 repatriations, without requesting any reimbursement. Similarly, in the early days of the COVID-19 crisis in March 2020, the Ministry of Foreign Affairs set up a specific repatriation plan in cooperation with airlines to facilitate the return of tens of thousands of French citizens stuck abroad (mostly tourists and travelers).

In the same report, French authorities claim that no other country in Europe had such advanced and comprehensive system of social protection for citizens abroad. For instance, unlike most other EU Member States who do not have guaranteed minimum resources schemes for citizens abroad, France allows for individual applications for direct cash payments to welfare committees set up at the consular level (*conseil consulaire pour la protection et l’action sociale*, CCPAS).

France is also very active on the cultural front, though its extra-territorial cultural policies mainly target non-resident foreigners as part of its diplomatic policy. These extra-territorial cultural policies however indirectly benefit French citizens abroad. Three main cultural institutions are in charge of these policies. First, the French Institutes (*Institut Français*), which were set up in their current form in 2010, but build on a historic network of institutes of French culture that started at the beginning of the twentieth century. The 2010 reform aimed to create an institution more similar to the German Goethe Institute or the British Institute, perceived as more effective at the time. The French Institute is co-administered by the Ministry of Foreign Affairs and the Ministry of Culture. Its core mission is to promote French culture abroad. In the law, no mention is made of French citizens abroad and French institutes clearly do not specifically target them. However, French institutes are often vibrant cultural centres and the natural hub of French communities abroad. Second, the French Alliance (*Alliance Française*) was created in 1883 with the purpose of promoting the French language and culture abroad. The organisation does not specifically target French citizens abroad either. In fact, it primarily targets “foreigners” in their home country, although its numerous services are also used by French citizens and its centres abroad often are the epicentre of French diasporic communities. They allow French citizens abroad to maintain a close link with French culture and ensure its reproduction across generation (together with the French Institute, especially since both institutions often share the same premises). According to its website, the *Alliance Française* has 1,036 offices dispersed in 130 countries. The third cultural arm of France abroad is the Agency for the Teaching of the French Language Abroad (*Agence pour l’enseignement français à l’étranger,* AEFE). It is responsible for managing the extra-territorial network of French schools that are affiliated to the French Ministry of Education. According to a 2016 Government report, the AEFE included 495 schools and 342,000 pupils in 2015, 125,000 of which being French citizens.

Overall, when it comes to cultural policies abroad, language is a core dimension, as these three institutions ensure the diffusion of the French language through language courses organised by the *Alliance Française* and the pursuit of compulsory education in one of the 495 French schools administered by the AEFE. In the centres of *Alliance Francaise*, French citizens or their children do not enjoy any preferential treatment and must comply with the same conditions as foreigners who wish to learn French or take an examination to prove their linguistic ability for professional or educational purposes.

Next to cultural and educational policies, it is to be noted that, unlike other large emigration states, France does not have economic policies targeting citizens abroad beyond the signature of 115 bilateral treaties to avoid double taxation. However, the decision of the current Government to abrogate the Solidarity Tax on Wealth in 2017 (*impot de solidarité sur la fortune*) was framed as a necessary measure for stopping the flight of wealthy tax payers and encouraging wealthy expatriates to return. While it is unclear whether or not this reform is primarily targeting the French abroad, discussions around it revealed the authorities’ preoccupation for a certain type of financial elites among the French population abroad.

## Diaspora Policies and Social Protection in France

In this section, we look in more details into French policies towards citizens abroad with a focus on social protection and show that, compared to other EU Member States, France has a more proactive approach in this area.

### Unemployment

With regards to unemployment benefits, French citizens residing outside the EU are not entitled to unemployment benefits in France upon their return if they were not affiliated to the (public) insurance scheme (*caisse d’assurance chômage*) during their residence abroad.

They may, however, remain affiliated while abroad in two different ways. First, if their employer abroad chooses to affiliate them to the scheme by paying a monthly social security contribution. This is frequent for persons employed by state agencies or large companies and posted abroad under an “expatriate status”. Second, French citizens abroad may individually choose to remain affiliated with the French unemployment scheme by paying a monthly contribution. They must do so prior to their expatriation or within 12 months after they left the country. They are then entitled to French unemployment benefits in case of job loss abroad, provided they return to the country within 12 months.^6^

Among their prerogatives, Consular Councils may intervene on unemployment-related issues in their constituency. For instance, expatriates may, in principle, receive support for professional training (under exceptional circumstances taking into account their personal situation and the economic context in their country of residence). In practice, very few have been eligible for such scheme. In 2015, for instance, merely EUR 35,000 were spent in three African countries (Madagascar, Mali, and Senegal).^7^

### Health Care

A critical actor in France’s health policies towards citizens abroad is the Fund for French Abroad (*Caisse des Français de l’étranger*, CFE) that was created in 1978. Though formally autonomous from the French general health agency (Social Security), the CFE is oversighted by the Ministries of Social Security and Budget and regulated by the French social security code. Beyond health provisions guaranteed by EU social security cooperation, the CFE allows French citizens residing outside the EU to subscribe to three insurance schemes, covering health and maternity, invalidity, and work-related accidents, respectively.

Regarding the health and maternity insurance (*Assurance maladie-maternité*), citizens abroad may remain affiliated to the French social security through its dedicated agency specifically catering for the needs of expatriates. The CFE offers a variety of schemes covering specific risks and ensuring coverage both in the country of residence as well as during visits/stays in France, under similar conditions as those provided by social security for residents (e.g. specific reimbursement rates, list of medicines covered by insurance, etc.). The monthly payment for the coverage varies according to several criteria – chiefly income and age, with preferential fees for the youth, the elderly, and families – thus mirroring the means-tested social policy scheme in the homeland. Once the person is affiliated, the CFE covers health-related expenses in the country of residence, with country-specific limitations and thresholds.

Second, the invalidity insurance scheme is subject to specific criteria: be less than 60 years old at the time of the contract; be formally declared as invalid (at least 2/3rd invalidity) and be able to justify a loss of income resulting from the person’s invalidity. Eligible individuals who subscribed to the insurance are entitled to benefits both in the country of residence and during their short stay in France. The exact amount and benefits are subject to country-specific limitations and thresholds.

Third, the CFE also provides insurance schemes covering work-related accidents, but citizens are also encouraged to contract a complementary (private) insurance to cover residual/extra health-related costs. The CFE has a formal partnership with private insurers either exclusively specialised in expatriates or with a dedicated branch for citizens abroad.^8^

### Pensions

Beyond the EU framework, recipients of French pensions are able to receive it while residing abroad by submitting once a year a life certificate (*certificat d’existence*) to pension authorities in France. The certificate must be signed by the consulate, embassy, or diplomatic office in the country of residence. Since 1 January 2018, the procedure may be done electronically.

French workers residing outside the EU are also able to remain affiliated to the general pension scheme. The old-age insurance (*Assurance Vieillesse*) for citizens abroad is administered by the CFE, which acts as an intermediary by managing the payments made by citizens abroad and transferring them to the insurance scheme for residents (*Caisse Nationale d’Assurance Vieillesse*), which is itself a branch of the French social security.

Lastly, citizens residing abroad have also access to a series of means-tested cash benefits. In particular, the “solidarity benefits for the elderly” (*Allocation de solidarité en faveur des personnes agées,* AS) is a benefit paid on a monthly basis to persons above 65 years old who meet certain economic conditions. This policy primarily targets resident citizens and foreigners who have worked and are retired in France. However, French citizens abroad are also eligible under specific conditions. For non-residents, individual applications are assessed by the Consular Council for social protection and aid located in the beneficiary’s country of residence (see details above regarding the *Conseil consulaire pour la protection et l’action sociale,* CCPAS).

### Family-Related Benefits

Beyond what is provided by EU regulations, no specific policy exists on the exportability of child benefits and family-related benefits do not formally fall under the competence of Consular Councils.^9^ Nonetheless, two related policies are worth being mentioned here. First, in the area of maternity benefits, French women giving birth abroad who subscribed to the health-maternity insurance scheme of the Fund for French Abroad (*Assurance santé-maternité de la caisse des Français de l’étranger*) are entitled to a *per diem* for maternity leave benefits, as well as coverage of a series of birth-related expenses.^10^ Second, in the area of education, there is a means-tested scholarship scheme for children in age of schooling called “Means-Tested Schooling Aid” (*Aide à la scolarité sous conditions de ressources*). This scheme is related to recent transformations in French policies in the area of education for French abroad. Until 2012, the tuition fees for attending a French school homologated by the French Department of Education – which vary greatly across countries, e.g. from circa 300 euros in Madagascar up to circa 20,000 euros in New York city –, were partly waived for the children of French citizens. This measure, introduced under the presidency of Nicolas Sarkozy, was abrogated in 2012, with the election of François Hollande. In 2018, children of French citizens may still be entitled to a financial assistance in the form of a partial tuition waiver based on socio-economic considerations. In order to be eligible for a scholarship, applicants must be registered in the consular registry of the country of schooling and meet certain income requirements (which vary across countries).^11^

### Economic Hardship

Similarly to other EU Member States, the French minimum income scheme (*Revenu de Solidarité Active,* RSA) is in principle reserved to residents, although beneficiaries may keep receiving the RSA should their stay abroad not exceed 3 months.^12^ Yet, when it comes to cash benefits for citizens abroad in situation of hardship, French policies are significantly different from what can be observed in the other case studies discussed in this volume as they go beyond schemes designed for citizens temporarily abroad who face exceptional circumstances.

Available cash payments cover a variety of circumstances and potential beneficiaries must apply to the Consular Council for social protection and aid (*Conseil consulaire pour la protection et l’action sociale”,* CCPAS) in the consulate of their consular constituency of residence. In each consulate, a civil servant usually occupies the post of delegate to social protection (*délégué à la protection sociale*). The CCPAS is composed of one or several consular civil servants, consular councillors (elected every 6 years), and representatives of civil society associations and stakeholders. It meets several times a year (the exact number of yearly sessions varies across consulates) and is in charge of determining individual applicants’ eligibility to a specific scheme, as well as the amount and length of the benefit, if applicable. In addition to Consular Councils abroad, there is also a Permanent Commission for the social protection of French citizens abroad in the Senate (*Commission permanente pour la protection sociale des Français de l’étranger*), created in 1992. Its role is essentially legislative: it does not assess individual cases, but it rather sets the general norms, together with the National Assembly.

There are four types of cash benefits available to citizens abroad in situation of economic hardship. First, there is the fixed-term social allowance (*Allocation à durée déterminée,* ADD) which is a time-limited financial assistance to French persons abroad who meet certain income requirements and find themselves “in distress”. Second, there is the child-specific scheme called *Secours mensuels spécifiques enfants* (SMSE) which also provides one-time extraordinary payment to children “in distress”. Third, there is a discretionary scheme called Occasional aids (*Secours occasionnels)* that covers expenses to French citizens facing exceptional circumstances abroad. Fourth, there is a scheme for non-registered citizens abroad and prisoners called “exceptional aids” (*Aides exceptionelles)* which is also a one-off payment made in exceptional circumstances.

In addition to those policies directly managed by consulates, France also supports associations that provide assistance to French citizens abroad. Such scheme is similar to the practice of other states such as Ireland that privilege delegating assistance missions to associations (see Hickman in this volume). Indeed, France provides funding to the so-called “Local Associations of assistance and solidarity” (*Organismes locaux d’entraide et de solidarité)*. These non-profit charities are located and operate exclusively abroad. Their purpose is to provide assistance to French citizens facing particular hardship, and whose situation does not fall under the remit of the CCPAS. Additionally, they can also provide benefits in-kind to persons receiving cash benefits from French authorities.

Finally, France also subsidises a homeland association that provides assistance to returnees, called France-Horizon. This association, originally created in 1940 to assist displaced French citizens during the war, now provides social assistance to a variety of vulnerable populations, including migrants, asylum seekers, and returnees facing exceptional circumstances.^13^

## Conclusions

In this chapter, we have shown that France is not only a country of immigration, but also one of emigration. French authorities have developed a wide range of policies that target a growing population of expatriates. In the area of electoral rights, culture, and social protection, the analysis of the French case showed a relatively stronger level of engagement compared to the other cases studied in this volume (Lafleur and Vintila this volume).

As far as social rights are concerned, a recent report of the Ministry of Europe and Foreign Affairs claims that France has the most advanced framework of social protection for its citizens abroad in Europe,^14^ an assertion which is corroborated by the comparative findings in the present volume (Lafleur and Vintila this volume). In one of the few comparative overviews of diaspora policies in Europe, Weinar found that France’s extensive diaspora policy “reflects, on one hand, the French state model in which the state is trusted and expected to support citizens in all instances, and on the other, the French view that the transnational mobility of its citizens is an opportunity to be used for the benefit of the state” (Weinar 2017: 2234–2235). In the past 30 years, the French welfare state has become increasingly de-territorialised due to the twin pressure of the EU integration process and domestic reforms in a context of welfare retrenchment. As a result, while residence has remained a central criteria for welfare eligibility, it has been loosened, both with regards to non-citizen residents and non-resident citizens (Isidro and Math 2020). The process has been uneven across policy areas, as this chapter has shown.

To understand France’s diaspora engagement policy, one must look at both institutional and ideational aspects. First, France has a highly egalitarian conception of citizenship, which is a constitutive element of the Republican pact, and has become increasingly disconnected from residence in the country. It is particularly true with regards to political rights, as the French national franchise does not dilute the voice of expatriates through malapportionment, but ensures equal representation in the Parliament, compared to domestic constituencies (Arrighi 2014, 2018). It is also embodied in a highly redistributive welfare state, which has been gradually expanded to citizens abroad. Second, France’s imperial past and the circumstances of decolonization need to be taken into account when looking at its engagement with citizens abroad. In material terms, France’s vast diplomatic network is the direct institutional legacy of its past as a prominent European and colonial power. A variety of policies directed to the diaspora today can be traced back in one way or another to colonial arrangements. Again, this is particularly significant in the electoral realm. Unlike the UK, France granted political representation to most of its colonial territories in the Parliament and, after independence, those individuals who retained their French citizenship were granted direct and discrete representation in the Parliament. Third, in ideational terms, France’s diaspora is essentially driven by its desire to project its power and prestige abroad, in an effort to convey the image of a super-power, both internally, to the French population, and externally, to the rest of the world. Needless to say, the myth of France’s omnipotence has become increasingly hard to sustain. The discrepancy between an idea of France which, as de Gaulle, famously said, “cannot be France without ‘grandeur’”, and the reality of a medium-sized power, is also reflected in a diaspora policy characterized by ambitious policy aims, and relatively modest policy outcomes.

## Footnotes

1 Sections of this chapter that concern the political participation and representation of French citizens abroad have been published in a report authored by Jean-Thomas Arrighi in the framework of the FAIREU H2020 Research project (Arrighi J.-T. (2018) “Report on political participation of mobile EU citizens: FRANCE” Accessible at https://faireu.ecas.org/wp-content/uploads/2019/02/FAIREU_France.pdf).

2 In the introductory chapter of this volume, the analysis of the French case is based on OECD data, which includes the US instead of Germany among the top five destination countries of the French diaspora.

3 *Ministère de l’Europe et des Affaire Etrangères* (2019). *Rapport sur la situation des Français établis hors de France*. http://www.assemblee-afe.fr/IMG/pdf/rapport_2019_-_version_finale_pdf.pdf. Accessed 05 May 2020.

4 The list of extra-territorial federations for mainstream parties can be consulted online, as follows: *Les Républicains* (LR, mainstream right, https://www.republicains.fr/federation); *Le Parti Socialiste* (PS, mainstream left, http://ffe-ps.org/); *La république en Marche* (LREM, centre, https://en-marche.fr/le-mouvement/la-carte). In the latest round of elections (June 2017), the results were as follows: 10 seats out of 11 for LREM or parties belonging to the presidential coalition, such as the centre party MODEM; one seat for a centre-left candidate, who was not running under the PS banner. See: https://www.diplomatie.gouv.fr/fr/services-aux-citoyens/droit-de-vote-et-elections-a-l-etranger/resultats-des-elections/article/elections-legislatives-resultats-pour-les-francais-de-l-etranger. All links were accessed on 18 August 2018.

5 For a comprehensive overview of non-resident citizens’ electoral rights in France, see Arrighi (2014). Electronic voting was first experimented in the 2012 parliamentary elections. It was cancelled shortly before the 2017 elections. Due to fears of “cyber-attacks”, dixit the Ministry of Interior. See: https://www.lemonde.fr/pixels/article/2017/03/06/legislatives-le-gouvernement-ne-recourra-pas-au-vote-electronique-pour-les-francais-de-l-etranger-pour-des-raisons-de-securite_5090026_4408996.html. Accessed 18 August 2018.

6 For more information, see the website of the French job centre (*Pôle emploi*): https://www.pole-emploi.fr/candidat/salarie-expatrie-hors-europe-@/article.jspz?id=60557. Accessed 18 August 2018.

7 See *Ministère de l’Europe et des Affaire Etrangères* (2019), Footnote 3.

8 More information at: https://www.cfe.fr/web/guest/adhesion-accident-travail-salarie. Accessed 18 August 2018.

9 See art. 3, *LOI n° 2013-659 du 22 juillet 2013 relative à la représentation des Français établis hors de France*.

10 More information on maternity benefits under the CFE insurance scheme can be found at http://www.consulaires.com/maternite-a-letranger/. Different limitations and thresholds apply across host countries.

11 In application of articles D531-45 to D531-51 of the Education Code (*code de l’éducation*).

12 More information on eligibility conditions can be found on the Government’s website: https://www.service-public.fr/particuliers/vosdroits/F33739. Accessed 18 August 2018.

13 https://www.france-horizon.fr. Accessed 18 August 2018.

14 See *Ministère de l’Europe et des Affaire Etrangères* (2019), Footnote 3.
